# Supplementary material for: The Small RNA Universe of Capitella teleta
Source: Front Mol Biosci. 2022 Feb 25;9:802814. doi: 10.3389/fmolb.2022.802814 (PMC8915122; doi:10.3389/fmolb.2022.802814)
Supplement: Supplementary file 1 [file DataSheet1.ZIP › Supplement/known/CAPTEscaffold_777_28948.pdf]

Provisional ID	:	CAPTEscaffold_777_28948
Score total	:	2257873.7
Score for star read(s)	:	3.9
Score for read counts	:	2257863.7
Score for mfe	:	1.6
Score for randfold	:	1.6
Score for cons. seed	:	3
Total read count	:	4428714
Mature read count	:	4427448
Loop read count	:	0
Star read count	:	1266

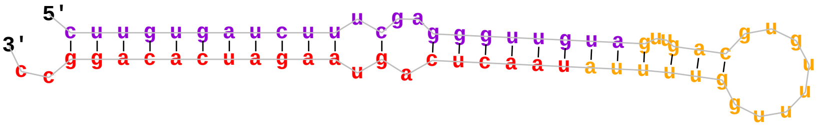

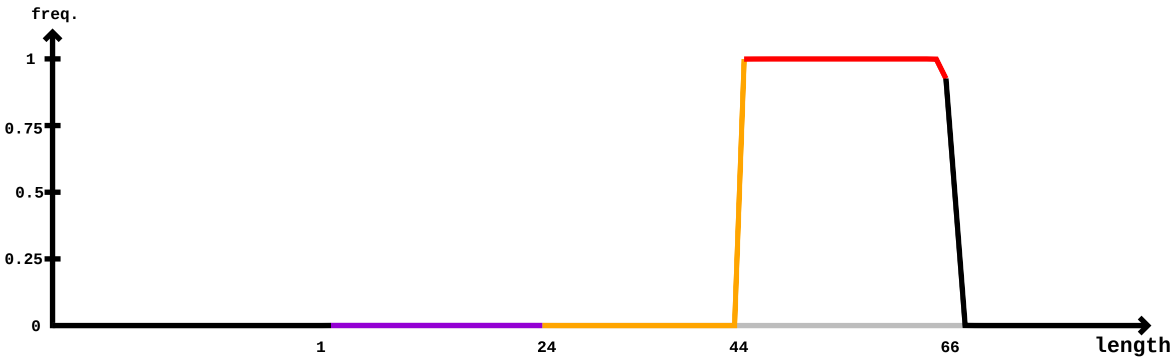


```
                                             Star                                       Mature
5'- uaacaguugcuagggaagcuugcgaaucuugugaucuuucgaggguuguaguugacguguuuugguuuuauaacucaguaagaucacaggccuugcauuuuucucggucuuc   -3'   obs
    uaacaguugcuagggaagcuugcgaaucuugugaucuuucgaggguuguaguugacguguuuugguuuuauaacucaguaagaucacaggccuugcauuuuucucggucuuc         exp
    uaacaguugcuagggaagcuugcgaaucuugugaucuuucgaggguuguaguugacguguuuugguuuuauaacucaguaagaucacaggccuugcauuuuucucggucuuc         known
    ........(((.((((((..(((((..(((((((((((.(..(((((((((..(((........))))))))))))).).)))))))))))..)))))..)))))))))....    reads   mm   sample
    .......ugcuagggaagcuugcgaau.....................................................................................    1       0    seq
    ...........................cuugugaucuuucgaggg...................................................................    10      0    seq
    ...........................cuugugaucuuucgagggu..................................................................    16      0    seq
    ...........................cuugugaucuuucgaggguu.................................................................    2       0    seq
    ...........................cuugugaucuuucgaggguug................................................................    67      0    seq
    ...........................cuugugaucuuucgaggguugu...............................................................    117     0    seq
    ...........................cuugugaucuuucgaggguugG...............................................................    1       1    seq
    ...........................cuugugaucCuucgaggguugua..............................................................    1       1    seq
    ...........................cuugugaucuuucgaggguugCa..............................................................    1       1    seq
    ...........................cCugugaucuuucgaggguugua..............................................................    1       1    seq
    ...........................cuugugauAuuucgaggguugua..............................................................    1       1    seq
    ...........................cuugugaucuuuUgaggguugua..............................................................    2       1    seq
    ...........................cuugugaucuuucgaggguuCua..............................................................    1       1    seq
    ...........................Uuugugaucuuucgaggguugua..............................................................    1       1    seq
    ...........................cAugugaucuuucgaggguugua..............................................................    2       1    seq
    ...........................cuAgugaucuuucgaggguugua..............................................................    1       1    seq
    ...........................cuugugaucuuucgaggguugua..............................................................    1013    0    seq
    ...........................cuugugaucuuucAaggguugua..............................................................    5       1    seq
    ...........................cuugugaucuuucgaggguuguU..............................................................    13      1    seq
    ...........................cuugugaucuuucgaggguuguaU.............................................................    7       1    seq
    ...........................cuugugaucuuucgaggguuguUgu............................................................    1       1    seq
    ...........................cuugugaucuuucgaggguuguagu............................................................    1       0    seq
    ..............................gugaucuuucgaggguugua..............................................................    2       0    seq
    ................................................................guuuuauaacucaguaagaucacaggcc....................    4       0    seq
    ...................................................................uuauaacucaguaagaucacaggcc....................    1       0    seq
    ...................................................................uuauaacucaguaagaucacaggcAu...................    1       1    seq
    ...................................................................Guauaacucaguaagaucacaggccu...................    3       1    seq
    ...................................................................uuauaacucaguaagaucacaggccu...................    97      0    seq
    ....................................................................Aauaacucaguaagaucacaggc.....................    1       1    seq
    ....................................................................uauaacucaguaagaucacaggcc....................    2       0    seq
    ....................................................................uCuaacucaguaagaucacaggcc....................    16      1    seq
    ....................................................................Aauaacucaguaagaucacaggccu...................    1       1    seq
```

Star   Mature

```
uaacaguugcuagggaagcuugcgaaucuugugaucuuucgaggguuguaguugacguguuuugguuuuauaacucaguaagaucacaggccuugcauuuuucucggucuuc
.....................................................................auaacucaguaagaucacagU......................	1	1	seq
.....................................................................auaacucaguaagaucacaggc.....................	19	0	seq
.....................................................................Cuaacucaguaagaucacaggc.....................	2	1	seq
.....................................................................auaacucaguaagaucacaggcA....................	1	1	seq
.....................................................................auaacucaguaagaucacaggcU....................	5	1	seq
.....................................................................auaacucagAaagaucacaggcc....................	1	1	seq
.....................................................................Uuaacucaguaagaucacaggcc....................	3	1	seq
.....................................................................auaacucaguaagaAcacaggcc....................	1	1	seq
.....................................................................auaacucaguaagaucacaggAc....................	1	1	seq
.....................................................................Nuaacucaguaagaucacaggcc....................	1	1	seq
.....................................................................Cuaacucaguaagaucacaggcc....................	80	1	seq
.....................................................................auaacucaguaagaucacaggcc....................	211	0	seq
.....................................................................auaacuAaguaagaucacaggccu...................	1	1	seq
.....................................................................auaacucaguaagaucacaggccu...................	129	0	seq
.....................................................................auaacucaguaagaucacaggccA...................	1	1	seq
.....................................................................Uuaacucaguaagaucacaggccu...................	1	1	seq
.....................................................................auaacucaguaagaucacaggccG...................	1	1	seq
.....................................................................Cuaacucaguaagaucacaggccu...................	36	1	seq
......................................................................uaacucaguaagaucaca........................	57	0	seq
......................................................................uaacucaguaagaucacag.......................	97	0	seq
......................................................................uaacucaguaagaucGcag.......................	2	1	seq
......................................................................uaUcucaguaagaucacag.......................	1	1	seq
......................................................................uaacucaguaagaGcacagg......................	2	1	seq
......................................................................uaacuAaguaagaucacagg......................	1	1	seq
......................................................................uaacucaguaagaucUcagg......................	1	1	seq
......................................................................uaacucaguaagaucacagU......................	53	1	seq
......................................................................uaacucaCuaagaucacagg......................	3	1	seq
......................................................................uaacucGguaagaucacagg......................	1	1	seq
......................................................................uaacAcaguaagaucacagg......................	7	1	seq
......................................................................uaacCcaguaagaucacagg......................	1	1	seq
......................................................................uaacucaguaaAaucacagg......................	1	1	seq
......................................................................uaacucaguaagaucacagA......................	28	1	seq
......................................................................Aaacucaguaagaucacagg......................	17	1	seq
......................................................................uaacuUaguaagaucacagg......................	4	1	seq
......................................................................uaacucaguaagauAacagg......................	1	1	seq
......................................................................Caacucaguaagaucacagg......................	2	1	seq
......................................................................uaacucaguaagaucacagC......................	4	1	seq
......................................................................uaacucaguaagaucacaCg......................	1	1	seq
......................................................................Gaacucaguaagaucacagg......................	2	1	seq
......................................................................uaacucaguaagaucacGgg......................	1	1	seq
......................................................................uaacucaguaagaucaAagg......................	1	1	seq
......................................................................uaacucaguaagaucacagg......................	3549	0	seq
......................................................................uaacucaguaagaAcacagg......................	1	1	seq
......................................................................uaGcucaguaagaucacagg......................	1	1	seq
......................................................................uaacucaguGagaucacagg......................	1	1	seq
......................................................................uaacucagAaagaucacagg......................	1	1	seq
......................................................................uaacucaAuaagaucacaggc.....................	37	1	seq
......................................................................uaacucaguaagaCcacaggc.....................	38	1	seq
......................................................................uaacucaguaagaucacaAgc.....................	74	1	seq
......................................................................uaacucaguaUgaucacaggc.....................	21	1	seq
......................................................................uaacucaguaaUaucacaggc.....................	20	1	seq
......................................................................uaacucaguaagaucacagNc.....................	1	1	seq
......................................................................uaacucaguaagaucacaggc.....................	314850	0	seq
......................................................................uaacucaguaagaucacaUgc.....................	25	1	seq
......................................................................uaacucUguaagaucacaggc.....................	24	1	seq
......................................................................uaacucaguaagaucUcaggc.....................	97	1	seq
......................................................................uaacucaguaagaucacaCgc.....................	10	1	seq
......................................................................uaacuGaguaagaucacaggc.....................	9	1	seq
......................................................................uaacucaguaagaucCcaggc.....................	5	1	seq
......................................................................uaacucaguaagaucacaggA.....................	883	1	seq
......................................................................uaacucaguaagaGcacaggc.....................	4	1	seq
......................................................................Naacucaguaagaucacaggc.....................	75	1	seq
......................................................................uaacucaguaagauAacaggc.....................	130	1	seq
......................................................................Caacucaguaagaucacaggc.....................	61	1	seq
......................................................................uaacCcaguaagaucacaggc.....................	37	1	seq
......................................................................uNacucaguaagaucacaggc.....................	11	1	seq
......................................................................uaaGucaguaagaucacaggc.....................	18	1	seq
......................................................................uaacucaCuaagaucacaggc.....................	43	1	seq
......................................................................uaacucaguaagaucaAaggc.....................	39	1	seq
......................................................................uaacucaguaagaNcacaggc.....................	1	1	seq
```

```
                                          Star                                    Mature
uaacaguugcuagggaagcuugcgaaucuugugaucuuucgaggguuguaguugacguguuuugguuuuauaacucaguaagaucacaggccuugcauuuuucucggucuuc
......................................................................uaacucaguaagaucacagUc.....................	45	1	seq
......................................................................uaacucaguaaCaucacaggc.....................	11	1	seq
......................................................................uaacucaUuaagaucacaggc.....................	21	1	seq
......................................................................uaacucGguaagaucacaggc.....................	60	1	seq
......................................................................uaacucaguaGgaucacaggc.....................	41	1	seq
......................................................................uaacucaguaagaucaUaggc.....................	30	1	seq
......................................................................uaacucagCaagaucacaggc.....................	27	1	seq
......................................................................uaacucaguaagauUacaggc.....................	112	1	seq
......................................................................uUacucaguaagaucacaggc.....................	3	1	seq
......................................................................uaacucaguGagaucacaggc.....................	85	1	seq
......................................................................uaacucaguaagUucacaggc.....................	28	1	seq
......................................................................uaacucaguaagaucacUggc.....................	18	1	seq
......................................................................uaacucaguaagaucacaggU.....................	380	1	seq
......................................................................uaacucaguaagaucGcaggc.....................	79	1	seq
......................................................................uaacucaguCagaucacaggc.....................	1	1	seq
......................................................................uaacucaguaagaucNcaggc.....................	1	1	seq
......................................................................uaacucCguaagaucacaggc.....................	8	1	seq
......................................................................uaacuAaguaagaucacaggc.....................	282	1	seq
......................................................................uaacucNguaagaucacaggc.....................	1	1	seq
......................................................................uaacucaguaagaAcacaggc.....................	51	1	seq
......................................................................uaacucaguaagGucacaggc.....................	52	1	seq
......................................................................uaacucaguaagauGacaggc.....................	18	1	seq
......................................................................uaacucaguaagaucacGggc.....................	72	1	seq
......................................................................Gaacucaguaagaucacaggc.....................	159	1	seq
......................................................................uaacGcaguaagaucacaggc.....................	11	1	seq
......................................................................uaacucaguaaAaucacaggc.....................	27	1	seq
......................................................................uaacucaguaagaucacaggG.....................	30	1	seq
......................................................................uaacucaguUagaucacaggc.....................	12	1	seq
......................................................................uaacuNaguaagaucacaggc.....................	14	1	seq
......................................................................uaacucaguaagaucaGaggc.....................	24	1	seq
......................................................................uaGcucaguaagaucacaggc.....................	40	1	seq
......................................................................uaacuUaguaagaucacaggc.....................	155	1	seq
......................................................................uaacucaguaCgaucacaggc.....................	3	1	seq
......................................................................uCacucaguaagaucacaggc.....................	1	1	seq
......................................................................uaaAucaguaagaucacaggc.....................	81	1	seq
......................................................................uaacucaguaagCucacaggc.....................	2	1	seq
......................................................................uaacucagGaagaucacaggc.....................	17	1	seq
......................................................................uaUcucaguaagaucacaggc.....................	24	1	seq
......................................................................uaacAcaguaagaucacaggc.....................	328	1	seq
......................................................................uaacucaguaagaucacCggc.....................	5	1	seq
......................................................................Aaacucaguaagaucacaggc.....................	1994	1	seq
......................................................................uaacucaguNagaucacaggc.....................	2	1	seq
......................................................................uaacucaguaagaucacagAc.....................	29	1	seq
......................................................................uaacucagAaagaucacaggc.....................	114	1	seq
......................................................................uGacucaguaagaucacaggc.....................	46	1	seq
......................................................................uaacucaguaagaucacagCc.....................	49	1	seq
......................................................................uaaUucaguaagaucacaggc.....................	23	1	seq
......................................................................uaacucaguaagaucacaggN.....................	1	1	seq
......................................................................uaacucaguaagaucacaAgcc....................	854	1	seq
......................................................................uaacucaguCagaucacaggcc....................	15	1	seq
......................................................................uaacucaguaagaucaNaggcc....................	7	1	seq
......................................................................uaacucaguaagaucacaUgcc....................	159	1	seq
......................................................................Caacucaguaagaucacaggcc....................	498	1	seq
......................................................................uaacucaguUagaucacaggcc....................	223	1	seq
......................................................................uaGcucaguaagaucacaggcc....................	359	1	seq
......................................................................uaacucaguGagaucacaggcc....................	1381	1	seq
......................................................................uaacucaguaagaucacagCcc....................	223	1	seq
......................................................................uaacucaguaagaucacaCgcc....................	181	1	seq
......................................................................uaacucaguaagaucacUggcc....................	128	1	seq
......................................................................uaacucagNaagaucacaggcc....................	1	1	seq
......................................................................uaacucaguaagUucacaggcc....................	331	1	seq
......................................................................uaacucaguaagaNcacaggcc....................	3	1	seq
......................................................................uaNcucaguaagaucacaggcc....................	18	1	seq
......................................................................uaacucaguaaCaucacaggcc....................	117	1	seq
......................................................................uaacucaAuaagaucacaggcc....................	284	1	seq
......................................................................uaacCcaguaagaucacaggcc....................	426	1	seq
......................................................................uaacucaguaagaucGcaggcc....................	681	1	seq
......................................................................Aaacucaguaagaucacaggcc....................	19842	1	seq
......................................................................uaacucCguaagaucacaggcc....................	65	1	seq
......................................................................uaUcucaguaagaucacaggcc....................	338	1	seq
```

```
                                   Star                                          Mature
uaacaguugcuagggaagcuugcgaaucuugugaucuuucgaggguuguaguugacguguuuugguuuuauaacucaguaagaucacaggccuugcauuuuucucggucuuc
......................................................................uaacucaguaGgaucacaggcc....................    555      1   seq
......................................................................uaacucaguaagaAcacaggcc....................    717      1   seq
......................................................................uaacucaUuaagaucacaggcc....................    299      1   seq
......................................................................uaacucaguaagaucacaggGc....................    242      1   seq
......................................................................uaacucaguaUgaucacaggcc....................    254      1   seq
......................................................................uaacucaguaagaucacagUcc....................    291      1   seq
......................................................................uaacucaguaaAaucacaggcc....................    414      1   seq
......................................................................uaacucaguaagaucNcaggcc....................    2        1   seq
......................................................................uaacucaguaagaucacGggcc....................    703      1   seq
......................................................................uaacucaguaagaucacaggcc....................    3441283  0   seq
......................................................................uaacucNguaagaucacaggcc....................    1        1   seq
......................................................................uaacucaguNagaucacaggcc....................    12       1   seq
......................................................................uaacucUguaagaucacaggcc....................    299      1   seq
......................................................................uaacucaCuaagaucacaggcc....................    456      1   seq
......................................................................uaacucaguaagaucaAaggcc....................    472      1   seq
......................................................................uaacucagCaagaucacaggcc....................    402      1   seq
......................................................................uaacucaguaagaCcacaggcc....................    488      1   seq
......................................................................uaacucaguaagauGacaggcc....................    122      1   seq
......................................................................uGacucaguaagaucacaggcc....................    542      1   seq
......................................................................uaacucaguaagaGcacaggcc....................    65       1   seq
......................................................................uaacucagGaagaucacaggcc....................    181      1   seq
......................................................................uaacNcaguaagaucacaggcc....................    1        1   seq
......................................................................uaacuAaguaagaucacaggcc....................    1893     1   seq
......................................................................uaacucaguaagaucCcaggcc....................    89       1   seq
......................................................................uNacucaguaagaucacaggcc....................    128      1   seq
......................................................................uaaUucaguaagaucacaggcc....................    192      1   seq
......................................................................uaacucaguaagauAacaggcc....................    1105     1   seq
......................................................................uaacGcaguaagaucacaggcc....................    155      1   seq
......................................................................uaacucGguaagaucacaggcc....................    828      1   seq
......................................................................uaaGucaguaagaucacaggcc....................    205      1   seq
......................................................................uaacucaguaagaucacCggcc....................    62       1   seq
......................................................................uaacuUaguaagaucacaggcc....................    2155     1   seq
......................................................................uUacucaguaagaucacaggcc....................    34       1   seq
......................................................................uaacucaguaNgaucacaggcc....................    2        1   seq
......................................................................uaacucaguaCgaucacaggcc....................    29       1   seq
......................................................................uaacucagAaagaucacaggcc....................    1271     1   seq
......................................................................uaacucaguaagaucacaggcU....................    6429     1   seq
......................................................................uaacucaguaaUaucacaggcc....................    225      1   seq
......................................................................uaaAucaguaagaucacaggcc....................    1161     1   seq
......................................................................uaacucaguaagaucacaggcA....................    4305     1   seq
......................................................................uaacucaNuaagaucacaggcc....................    3        1   seq
......................................................................uaacucaguaagauUacaggcc....................    1663     1   seq
......................................................................uaacucaguaagaucacaggcG....................    253      1   seq
......................................................................uaacucaguaagaucacaggAc....................    1008     1   seq
......................................................................uaacucaguaagaucaUaggcc....................    418      1   seq
......................................................................uaacucaguaagGucacaggcc....................    572      1   seq
......................................................................uaacAcaguaagaucacaggcc....................    4060     1   seq
......................................................................uaacucaguaagCucacaggcc....................    26       1   seq
......................................................................Naacucaguaagaucacaggcc....................    899      1   seq
......................................................................uaacucaguaagaucaGaggcc....................    126      1   seq
......................................................................uaacuGaguaagaucacaggcc....................    149      1   seq
......................................................................uaacucaguaagaucacagNcc....................    3        1   seq
......................................................................uaacucaguaagaucacaggUc....................    410      1   seq
......................................................................Gaacucaguaagaucacaggcc....................    1870     1   seq
......................................................................uaacucaguaagaucacagAcc....................    508      1   seq
......................................................................uaCcucaguaagaucacaggcc....................    37       1   seq
......................................................................uaacucaguaagaucacaggcN....................    3        1   seq
......................................................................uaacuNaguaagaucacaggcc....................    50       1   seq
......................................................................uaacucaguaagaucUcaggcc....................    612      1   seq
......................................................................uaacucaguaagaucacaggNc....................    8        1   seq
......................................................................uCacucaguaagaucacaggcc....................    14       1   seq
......................................................................uaacucaguaagaucacCggccu...................    11       1   seq
......................................................................uaacucaguaagaucacaggccN...................    13       1   seq
......................................................................uaacucGguaagaucacaggccu...................    137      1   seq
......................................................................uaacucaguaagaucGcaggccu...................    121      1   seq
......................................................................uaaAucaguaagaucacaggccu...................    152      1   seq
......................................................................uNacucaguaagaucacaggccu...................    16       1   seq
......................................................................uaacucaguaagaucacaggccG...................    2885     1   seq
......................................................................uaacucagAaagaucacaggccu...................    196      1   seq
......................................................................uaacucaguaagCucacaggccu...................    7        1   seq
```

Star Mature

```
uaacaguugcuagggaagcuugcgaaucuugugaucuuucgaggguuguaguugacguguuuugguuuuauaacucaguaagaucacaggccuugcauuuuucucggucuuc
......................................................................uaNcucaguaagaucacaggccu...................	2	1	seq
......................................................................uaacucaguaagaNcacaggccu...................	1	1	seq
......................................................................Naacucaguaagaucacaggccu...................	172	1	seq
......................................................................uaacucaguaagaucacGggccu...................	112	1	seq
......................................................................uaacucaguaagaucacUggccu...................	22	1	seq
......................................................................Gaacucaguaagaucacaggccu...................	256	1	seq
......................................................................uaacucaguaagaucCcaggccu...................	15	1	seq
......................................................................uaacucaguaagaucacaUgccu...................	23	1	seq
......................................................................uaacucaguaagaucacaggccC...................	1608	1	seq
......................................................................uaacucaguaagaucacaggcGu...................	35	1	seq
......................................................................uaacucaguaagaucacaggcUu...................	185	1	seq
......................................................................uaUcucaguaagaucacaggccu...................	62	1	seq
......................................................................uaacucagGaagaucacaggccu...................	27	1	seq
......................................................................uaacucaguaaCaucacaggccu...................	19	1	seq
......................................................................uaacucaguaaUaucacaggccu...................	34	1	seq
......................................................................uaaUucaguaagaucacaggccu...................	34	1	seq
......................................................................uaacucaguaagaucacagUccu...................	35	1	seq
......................................................................uaacucaguaagaucacaggAcu...................	161	1	seq
......................................................................uaacucaguaagaucacaggcNu...................	1	1	seq
......................................................................uaacucaguaUgaucacaggccu...................	37	1	seq
......................................................................uaacuGaguaagaucacaggccu...................	17	1	seq
......................................................................uaacuNaguaagaucacaggccu...................	7	1	seq
......................................................................uaacCcaguaagaucacaggccu...................	50	1	seq
......................................................................uaacucaguaagaucacaggcAu...................	278	1	seq
......................................................................uaaGucaguaagaucacaggccu...................	19	1	seq
......................................................................uaacucaguaagaucacaAgccu...................	108	1	seq
......................................................................uaacucaguaagGucacaggccu...................	108	1	seq
......................................................................uaacucaguaCgaucacaggccu...................	5	1	seq
......................................................................uaacucaguUagaucacaggccu...................	16	1	seq
......................................................................uaacucaguaagaucacagCccu...................	46	1	seq
......................................................................uaacucaguaagaucacaggccu...................	566807	0	seq
......................................................................uaacucaguaagaCcacaggccu...................	71	1	seq
......................................................................uaacucaguaagaucacaCgccu...................	28	1	seq
......................................................................uaacucaguaagaucacaggNcu...................	2	1	seq
......................................................................uaacucaguaagaucaNaggccu...................	1	1	seq
......................................................................uaacGcaguaagaucacaggccu...................	25	1	seq
......................................................................uGacucaguaagaucacaggccu...................	85	1	seq
......................................................................uaacucUguaagaucacaggccu...................	47	1	seq
......................................................................Caacucaguaagaucacaggccu...................	67	1	seq
......................................................................uaacucaguGagaucacaggccu...................	226	1	seq
......................................................................uaacucaAuaagaucacaggccu...................	49	1	seq
......................................................................uaGcucaguaagaucacaggccu...................	74	1	seq
......................................................................uaacucaCuaagaucacaggccu...................	60	1	seq
......................................................................uaacucaguCagaucacaggccu...................	4	1	seq
......................................................................uaacucaguaagaucaGaggccu...................	40	1	seq
......................................................................uCacucaguaagaucacaggccu...................	4	1	seq
......................................................................uaacucaguNagaucacaggccu...................	4	1	seq
......................................................................uaacuAaguaagaucacaggccu...................	288	1	seq
......................................................................uaacucaguaagaucacaggGcu...................	37	1	seq
......................................................................uaacucaguaagaucUcaggccu...................	100	1	seq
......................................................................uaacucaguaagaGcacaggccu...................	18	1	seq
......................................................................uaacucaguaagauGacaggccu...................	27	1	seq
......................................................................uaacucaguaagauAacaggccu...................	195	1	seq
......................................................................Aaacucaguaagaucacaggccu...................	3229	1	seq
......................................................................uaacucaguaagaucacaggUcu...................	64	1	seq
......................................................................uaacucaguaagaucacaggccA...................	10294	1	seq
......................................................................uaacucCguaagaucacaggccu...................	10	1	seq
......................................................................uaacucaguaagaucacagAccu...................	90	1	seq
......................................................................uaCcucaguaagaucacaggccu...................	4	1	seq
......................................................................uaacucaguaagaucaAaggccu...................	64	1	seq
......................................................................uaacucaguaaAaucacaggccu...................	62	1	seq
......................................................................uaacucaguaagaucacagNccu...................	1	1	seq
......................................................................uaacucaguaagauUacaggccu...................	177	1	seq
......................................................................uaacAcaguaagaucacaggccu...................	567	1	seq
......................................................................uaacucaUuaagaucacaggccu...................	41	1	seq
......................................................................uaacuUaguaagaucacaggccu...................	321	1	seq
......................................................................uUacucaguaagaucacaggccu...................	7	1	seq
......................................................................uaacucaguaagUucacaggccu...................	62	1	seq
......................................................................uaacucaguaagaucaUaggccu...................	77	1	seq
......................................................................uaacucNguaagaucacaggccu...................	1	1	seq
```

Star **Mature**

```
uaacaguugcuagggaagcuugcgaaucuugugaucuuucgaggguuguaguugacguguuuugguuuuauaacucaguaagaucacaggccuugcauuuuucucggucuuc
......................................................................uaacucaguaagaAcacaggccu...................   116    1    seq
......................................................................uaacucaguaGgaucacaggccu...................   66     1    seq
......................................................................uaacucagCaagaucacaggccu...................   62     1    seq
......................................................................Aaacucaguaagaucacaggccuu..................   6      1    seq
......................................................................uaacucaguaagUucacaggccuu..................   1      1    seq
......................................................................uaacuAaguaagaucacaggccuu..................   1      1    seq
......................................................................uaacucaguaagaucacaggccuA..................   151    1    seq
......................................................................uaacucagCaagaucacaggccuu..................   1      1    seq
......................................................................Gaacucaguaagaucacaggccuu..................   1      1    seq
......................................................................uaacucaguaagaucacaggccuu..................   1149   0    seq
......................................................................uaacucaguaagaucacaggccuG..................   3      1    seq
......................................................................uaacucaguaagaucacaggccCu..................   8      1    seq
......................................................................uaacucaguaagaucacaggcAuu..................   47     1    seq
......................................................................uaacucaguaagaucacaggccAu..................   372    1    seq
......................................................................uaacucagAaagaucacaggccuu..................   1      1    seq
......................................................................uaacAcaguaagaucacaggccuu..................   1      1    seq
......................................................................uaacucaguaagaucacaggcUuu..................   1      1    seq
......................................................................uaacucaguaagaucacaggccuC..................   1      1    seq
......................................................................uaacucaguaagaucacaggccuug.................   3      0    seq
......................................................................uaacucaguaagaucacaggccuuA.................   210    1    seq
......................................................................uaacucaguaagaucacaggccuuU.................   313    1    seq
.......................................................................Uacucaguaagaucacaggc.....................   19     1    seq
.......................................................................aacucaguaagaucacaggc.....................   75     0    seq
.......................................................................aacucaguaagaucGcaggc.....................   1      1    seq
.......................................................................Gacucaguaagaucacaggcc....................   2      1    seq
.......................................................................aacucaguaagaucacaggcc....................   750    0    seq
.......................................................................aacuAaguaagaucacaggcc....................   1      1    seq
.......................................................................Nacucaguaagaucacaggcc....................   1      1    seq
.......................................................................aacucaguaagaucacaggcU....................   2      1    seq
.......................................................................aacucaguaagaucacaggcA....................   1      1    seq
.......................................................................Uacucaguaagaucacaggcc....................   223    1    seq
.......................................................................aacucaguaagaucacaggAc....................   1      1    seq
.......................................................................aacucaguaagaucacaAgccu...................   1      1    seq
.......................................................................Uacucaguaagaucacaggccu...................   36     1    seq
.......................................................................Gacucaguaagaucacaggccu...................   1      1    seq
.......................................................................aacucaguaagaucacaggccG...................   1      1    seq
.......................................................................aacucaguaagaucacaggccA...................   4      1    seq
.......................................................................aacucaguaagaucacaggccu...................   333    0    seq
........................................................................acucaguaagaucacaggc.....................   8      0    seq
........................................................................acucaguaagaucUcaggcc....................   1      1    seq
........................................................................acucaguaagaucacaggcc....................   376    0    seq
........................................................................acAcaguaagaucacaggcc....................   1      1    seq
........................................................................acucaguaagauAacaggcc....................   1      1    seq
.........................................................................cucaguaagaucacaggcc....................   179    0    seq
.........................................................................cucGguaagaucacaggcc....................   1      1    seq
..........................................................................ucaguaagaucacaggcc....................   124    0    seq
..........................................................................ucaguaagaucacaggccu...................   7      0    seq
..........................................................................ucaguaagaucacaggccG...................   1      1    seq
...........................................................................caguaagaucacaggccG...................   1      1    seq
...........................................................................caguaagauGacaggccu...................   1      1    seq
...........................................................................caguaagaucacaggccu...................   37     0    seq
............................................................................aguaagaucacaggccuugcauu.............   1      0    seq
............................................................................................uugcauuuuucucggucu..   14     0    seq
............................................................................................uugcauuuuucucggucuu.   2      0    seq
............................................................................................uugcauuuuucucggucuuc   1      0    seq
```
